# Association between modifiable lifestyle pattern and periodontitis: a cross-sectional study based on NHANES

**DOI:** 10.1186/s12903-024-04207-8

**Published:** 2024-05-21

**Authors:** Jianlin Lin, Tao Pei, Hongyu Yang

**Affiliations:** 1 Stomatological Center, Peking University Shenzhen Hospital, Lianhua Road, FutianDistrict, Shenzhen, Guangdong Province 518036 China; 2 Shenzhen Clinical Research Center for Oral Diseases, Shenzhen, Guangdong Province China; 3 Guangdong Provincial High-level Clinical Key Specialty, Shenzhen, Guangdong Province China; 4 Guangdong Province Engineering Research Center of Oral Disease Diagnosis and Treatment, Shenzhen, Guangdong Province China; 5 The Institute of Stomatology, Peking University Shenzhen Hospital, Shenzhen Peking University The Hong Kong University of Science and Technology Medical Center, Shenzhen, Guangdong China

**Keywords:** Periodontitis, Lifestyle, Latent class analysis, Risk factors

## Abstract

**Background:**

Periodontitis can be avoided with a healthy lifestyle. However, studies have only looked at one lifestyle, ignoring the connection between lifestyle patterns and periodontitis. The purpose of this study was to look at the association between modifiable lifestyle patterns and periodontitis.

**Methods:**

Data were obtained from the National Health and Nutrition Examination Survey in 2009–2010 and 2011–2012. Smoke, drink, exercise, sleep duration, oral exams, and self-rated oral health were all lifestyle factors. The CDC/AAP classification/case definition was used to evaluate periodontitis. Drawing upon latent class analysis, distinct patterns of lifestyle were identified, with each participant exclusively affiliated with a single pattern. The association between lifestyle classes and periodontitis was then examined using ordinal logistic regression.

**Results:**

4686 (52%) of the total 9034 participants, with a mean age of 54.08, were women. Three lifestyle latent classes were found by fitting 2–10 models: “Class 1” (52%), " Class 2” (13%), and " Class 3” (35%). The “Class 1” displayed a prevalence of oral examination (75%), favorable self-rated oral health (92%), and engagement in physical activity (50%). The ‘Class 2’ exhibited the lowest alcohol consumption (64%) and smoking rates (73%) but the highest prevalence of physical inactivity (98%). The ‘Class 3’ showed a tendency for smoking (72%), alcohol consumption (78%), shorter sleep duration (50%), absence of oral examinations (75%), and suboptimal self-rated oral health (68%). The influencing variables for the latent classes of lifestyle were age, education, and poverty level. Periodontitis risk may rise by 24% for each additional unhealthy lifestyle practiced by participants (OR = 1.24, 95% CI: 1.18–1.31). The ‘Class 3’ (OR = 1.80, 95% CI: 1.52–2.13) had a greater risk of periodontitis compared to the ‘Class 1’.

**Conclusions:**

Our analysis revealed that unhealthy lifestyle patterns are associated with periodontitis. These different lifestyle patterns need to be taken into account when developing public health interventions and clinical care.

**Supplementary Information:**

The online version contains supplementary material available at 10.1186/s12903-024-04207-8.

## Introduction

A chronic inflammatory illness affecting the tissues supporting the teeth, periodontitis is brought on by bacterial plaque [1]. Periodontitis is thought to affect 10% of people worldwide, whereas it affects approximately 42.2% of adults in the United States [2]. Diabetes, cognitive impairment, and cardiovascular and cerebrovascular illnesses are associated with periodontitis that pose a severe threat to public health globally [3–5]. The results of numerous research on the association between lifestyle and periodontitis suggest that decisions made regarding smoking, drinking, physical activity, and sleep duration may have an impact on how the disease manifests and advances [6–8]. These data collectively imply that lifestyle may have an impact on periodontitis.

High-risk populations will benefit from cost-efficient periodontitis control techniques and successful lifestyle change programs. Adults (particularly the elderly) are at a 66% higher risk of dying from bad habits and lifestyles, such as smoking, not getting enough exercise, eating poorly, and consuming too much alcohol [9]. These undesirable practices frequently occur or cluster within people. The majority of studies to date have only been concerned with modifying one unhealthy behavior [6]. Single lifestyle choices may not have much of an impact on periodontitis and may not give us enough knowledge to design successful lifestyle therapies. For example, people who drink and smoke have a higher chance of developing periodontitis than those who do not. However, there may be other groups of people who do not consume alcohol or tobacco but never get a dental examination and who also have a higher risk of developing periodontitis. Studies have examined the connection between periodontitis and lifestyle scores (i.e. the number of healthy lifestyles), however, it is impossible to determine the health advantages of people who lead various healthy lifestyles but have a score of 1, nor is it possible to use tailored interventions [6]. Consequently, the discernment of patterns associated with the integration of different lifestyles is essential for the development of all-encompassing strategies to enhance the quality of life and forestall periodontitis.

While investigations have explored the association between lifestyle choices and periodontitis, conventional clustering and factor analysis have primarily focused on continuous variables. Consequently, they fall short in elucidating the intricate associations among categorical variables, resulting in limited interpretability. Latent class analysis (LCA) offers a statistical approach adept at addressing categorical variables [10], particularly as lifestyle measures often manifest as binary variables. LCA categorizes the population into subgroups based on the probability of individuals responding to the measured variables. LCA produces good interpretability and has been used in some studies to comprehend the various lifestyle patterns. For instance, a previous study based on diet, physical activity patterns, smoking, and blood pressure control of hypertensive patients, classified their lifestyles into three risk classes: low-risk class (I), intermediate-risk class (II), and high-risk class (III), and examined the patterns in relation to sociodemographic characteristics [11]. Another Israeli study divided lifestyle into three possible categories: “healthy,” “unhealthy,” and “mixed” based on individuals’ consumption of fruits and vegetables, smoking, physical activity, sleep, and vaccination [12]. These findings suggest that lifestyles have different latent class characteristics. By identifying these latent patterns of lifestyle, it is possible to make targeted comprehensive lifestyle improvement recommendations for different classes of participants. However, studies on the association between lifestyle and specific diseases, like cognitive impairment and debilitating conditions, have been undertaken [13]. No research has examined the connection between various lifestyle choices and the risk of periodontitis to this point.

To fill this gap, the primary aims of this study are: (1) to identify latent lifestyle classes of adults aged 18 years or more using a nationally representative population, and (2) to look at the association between latent lifestyle classes and periodontitis. The results of this study may provide evidence to support future interventional studies aimed at investigating whether targeting specific clusters of unhealthy behaviors could help reduce the prevalence of periodontitis.

## Methods

The STROBE (Strengthening the Reporting of Observational Studies in Epidemiology) criteria were used in this study to conduct cross-sectional research. (Additional file 1).

### Data source

Data for waves 2009–2010 and 2011–2012 of the National Health and Nutrition Examination Survey (NHANES) were gathered. Detailed information on NHANES has been presented in previous studies [14–16]. The study’s subjects had at least five full records of their lifestyles and were older than 18 years old. These records were used for latent class analysis. Subjects got a thorough dental health-periodontic examination as part of the NHANES, and all measurements were taken by the periodontal categorization methodology to investigate the association between lifestyle choices and periodontitis. The study had 9034 participants in all.

#### Lifestyle and lifestyle scores

Lifestyle is comprised of two components [17]: health behaviors and health beliefs. Health behaviors were assessed through five indicators: smoking, drinking, physical activity (PA), sleep duration, and oral examination. Health beliefs were assessed through one indicator: self-rated oral health. All variables were binary. Smoking was defined as current smoking or quitting for less than one year. Drinking was defined as greater than 12 drinks a year. Through a questionnaire based on the Physical Activity questionnaire, physical activity (PA) data was gathered. Participants disclosed how often and for how long they engaged in vigorous or moderate recreational activities, as well as vigorous or moderate job activities. According to previous NHANES recommendations, the following activities received MET scores: vigorous work-related activity, moderate work-related activity, walking or bicycling, vigorous leisure-time activity, and moderate leisure-time activity, which were given 8, 4, 4, 8, and 4 points, respectively [18]. The sum of weekly activity minutes was multiplied by the MET score for each activity to determine the total PA. The US PA guidelines stated that low-level PA was classified as 500 MET-min/week and high-level PA as 500 MET-min/week [19]. Insufficient sleep was defined as a sleep duration of fewer than 7 h per day. Oral examination asked participants if they attended oral health regularly. Participants were considered to have had an oral examination if they indicated engagement in procedures such as check-ups, examinations, or cleanings. Self-rated oral health was self-reported by participants as good or poor. We also calculated a lifestyle score, with one point for having poor health behavior and a score range of 0–6.

#### Periodontitis

Six locations per tooth are measured as part of the NHANES periodontal examination technique on 28 teeth. Based on clinical attachment loss (AL)and probing depth (PD), periodontitis is diagnosed. The term “periodontitis” was defined in this study using the CDC/AAP classification/case definition [20]. Patients with mild periodontitis have two proximal sites on distinct teeth that have PD less than 5 mm or two proximal sites with clinical AL less than 4 mm. Patients with moderate periodontitis have one or more of the following: two proximal sites with PD greater than 5 mm, one PD greater than 4 mm, or two proximal sites with clinical AL smaller than 3 mm in distinct teeth. A tooth with a PD of 5 mm or at least two teeth with an AL of greater than or equal to 6 mm indicates severe periodontitis. Participants without any of these symptoms were considered to be free of periodontitis.

#### Covariates

Potential confounders related to the association between lifestyle and periodontitis were included in multivariate models, thus covariates were also identified with investigators and categorized in a manner consistent with previous studies [21]. Covariates including age (< 55 years, ≥ 55 years), sex (male, female), years of education (< 12 years, 12 years, > 12 years) [22], marriage (no, yes), race (Mexican-American; white, non-Hispanic; black, non-Hispanic; and other), poverty level, BMI and diabetes. Poverty levels were classified into 3 categories according to the algorithm of previous studies: <1.35 classified as low, 1.35-2 classified as medium, and ≥ 2 classified as high [23]. Three categories of BMI were established: low (18.5 kg/m^2^), normal (18.5–25 kg/m^2^), and high (25 kg/m^2^) [24]. Participants were classified as having diabetes if they met any of the following criteria: (1) physician-diagnosed diabetes, (2) glycohemoglobin HbA1c(%) ≥ 6.5, (3) fasting glucose (mmol/l) ≥ 7.0, (4) random blood glucose (mmol/l) ≥ 11.1, or (5) two-hour OGTT blood glucose (mmol/l) ≥ 11.1 [25].

### Statistical analysis

For categorical variables, N (%) descriptors were employed. The chi-square test was utilized to assess disparities in baseline data among patients exhibiting distinct degrees of periodontitis. First, we used latent class analysis (LCA) to identify a number of lifestyle classes. LCA is a statistical method that classifies individuals into different groups based on the participants’ response patterns on lifestyle [10]. According to the likelihood of unique conditions of lifestyle variables in each latent class, lifestyle latent classes are labeled. LCA estimates the parameters using the expectation-maximization algorithm, which accepts the existence of missing values in the data. LCA were conducted, varying the number of classes from 2 to 10. The optimal number of latent classes was determined through a rigorous evaluation based on statistical metrics and interpretability. The optimal LCA was chosen using low values of Akaike’s information criterion (AIC), adjusted Bayesian information criterion (aBIC), Bayesian information criteria (BIC), and consistent Akaike’s information criterion (cAIC) [26]. The model’s simplicity and interpretability were taken into account in addition to the model fit indicators. Second, we looked at the basic characteristics of various lifestyle groups using weighted logistic regression models. In order to evaluate the association between lifestyle groups and the severity of periodontitis, weighted ordinal logistic regression models were employed. The two-year sample weights for each NHANES period were aggregated into 2009–2012 weights. This research also examined the correlation between lifestyle scores and the severity of periodontitis to compare with earlier findings. All statistical calculations were done in R. The ‘poLCA’ package was used to conduct the study on LCA. The threshold for statistical significance was *P* < 0.05.

## Results

### Sample characteristics

Of the 9034 participants included, a total of 4686 (52.40%) were female, with a mean age of 54.08 years, and 62.28% were currently married. Of the total participants, 2057 participants had no periodontitis, 154 had mild periodontitis, 306 had moderate periodontitis, and 6517 had severe periodontitis (Table 1). Univariate results showed that different degrees of periodontitis were associated with physical activity, sleep duration, oral examinations, and self-rated oral health. The median score for poor lifestyle was 2.

**Table 1:** Sociodemographic characteristics of sample grouped by periodontitis (N, %)

	Total	No	mild	moderate	severe		P-value
Age						< 0.001	
< 55 years	4719(56.19)	1245(63.91)	130(81.96)	235(69.13)	3109(51.73)		
≥55 years	4315(43.81)	812(36.09)	24(18.04)	71(30.87)	3408(48.27)		
**Sex**						**< 0.001**	
Men	4348(47.60)	888(43.68)	70(44.42)	176(58.73)	3214(48.58)		
Women	4686(52.40)	1169(56.32)	84(55.58)	130(41.27)	3303(51.42)		
**Education**						**0.01**	
<12	3432(33.11)	713(28.98)	45(24.96)	86(31.11)	2588(35.04)		
12	5602(66.89)	1344(71.02)	109(75.04)	220(68.89)	3929(64.96)		
>12						**< 0.001**	
**Current marital status**	2178(16.72)	395(11.97)	7(2.49)	55(13.44)	1721(19.16)		
No	1976(21.00)	346(14.61)	23(13.36)	58(17.65)	1549(23.82)		
Yes	4880(62.28)	1316(73.42)	124(84.15)	193(68.92)	3247(57.02)		
**Race**						**< 0.001**	
Mexican-American	1037(7.69)	221(6.63)	12(5.50)	55(13.49)	749(7.85)		
White, Non-Hispanic	3700(68.30)	947(73.25)	77(75.21)	103(60.12)	2573(66.65)		
Black, Non-Hispanic	2087(11.05)	410(9.39)	22(5.94)	49(8.96)	1606(11.96)		
Other	2210(12.96)	479(10.73)	43(13.35)	99(17.43)	1589(13.54)		
**Poverty level**						**< 0.001**	
Low	2857(21.61)	540(17.21)	30(12.52)	70(16.97)	2217(26.23)		
Median	2156(23.73)	426(21.62)	26(18.57)	80(26.70)	1624(27.17)		
High	3205(47.60)	909(61.16)	88(68.91)	131(56.33)	2077(46.59)		
**BMI**						0.35	
Low	123(1.10)	30(1.24)	1(0.93)	1(0.38)	91(1.11)		
Normal	2381(25.23)	575(27.35)	47(29.29)	85(26.51)	1674(24.68)		
High	6390(72.48)	1384(71.41)	105(69.78)	217(73.12)	4684(74.21)		
**Smoking**						**0.03**	
No	2055(23.84)	431(58.15)	25(61.42)	56(53.07)	1543(50.59)		
Yes	1990(21.59)	357(41.85)	18(38.58)	58(46.93)	1557(49.41)		
**Drinking**						0.30	
No	2289(20.05)	473(22.66)	38(24.41)	64(16.75)	1714(22.07)		
Yes	5734(70.79)	1168(77.34)	97(75.59)	202(83.25)	4267(77.93)		
**Physical activity**						0.14	
Yes	3277(41.19)	678(42.06)	69(45.81)	135(52.48)	2395(43.64)		
No	5112(53.24)	1119(57.94)	71(54.19)	140(47.52)	3782(56.36)		
**Sleep duration**						**< 0.001**	
Sufficient	5048(60.12)	1126(67.33)	99(75.68)	169(64.43)	3654(61.95)		
Insufficient	3338(34.28)	670(32.67)	41(24.32)	106(35.57)	2521(38.05)		
**Oral examination**						**< 0.001**	
Yes	4167(54.19)	1100(67.83)	117(83.90)	181(68.49)	2769(52.52)		
No	4181(39.87)	689(32.17)	23(16.10)	91(31.51)	3378(47.48)		
**Self-rated oral health**						**< 0.001**	
Good	5528(67.71)	1314(80.10)	127(92.85)	211(79.43)	3876(67.62)		
Poor	2861(26.72)	483(19.90)	13(7.15)	64(20.57)	2301(32.38)		
**Lifestyle scores [Q1,Q3]**	2.00(1.00,3.00)	2.00(1.00,3.00)	1.00(0.00,2.00)	2.00(1.00,3.00)	2.00(1.00,3.00)	**< 0.001**	
**Diabetes**						**< 0.001**	
No	6289(74.30)	1488(78.20)	127(84.82)	244(79.44)	4430(72.85)		
Yes	2697(25.13)	553(21.80)	25(15.18)	59(20.56)	2060(27.15)		

*Notes* This table reported counts (absolute frequencies - N) and weighted relative frequencies (proportion - %)

Pearson chi-square tests for categorical variables

*Abbreviations* CI, confidence interval; OR, odds ratios

### Identification of the latent class of lifestyle

**Fig. 1:**
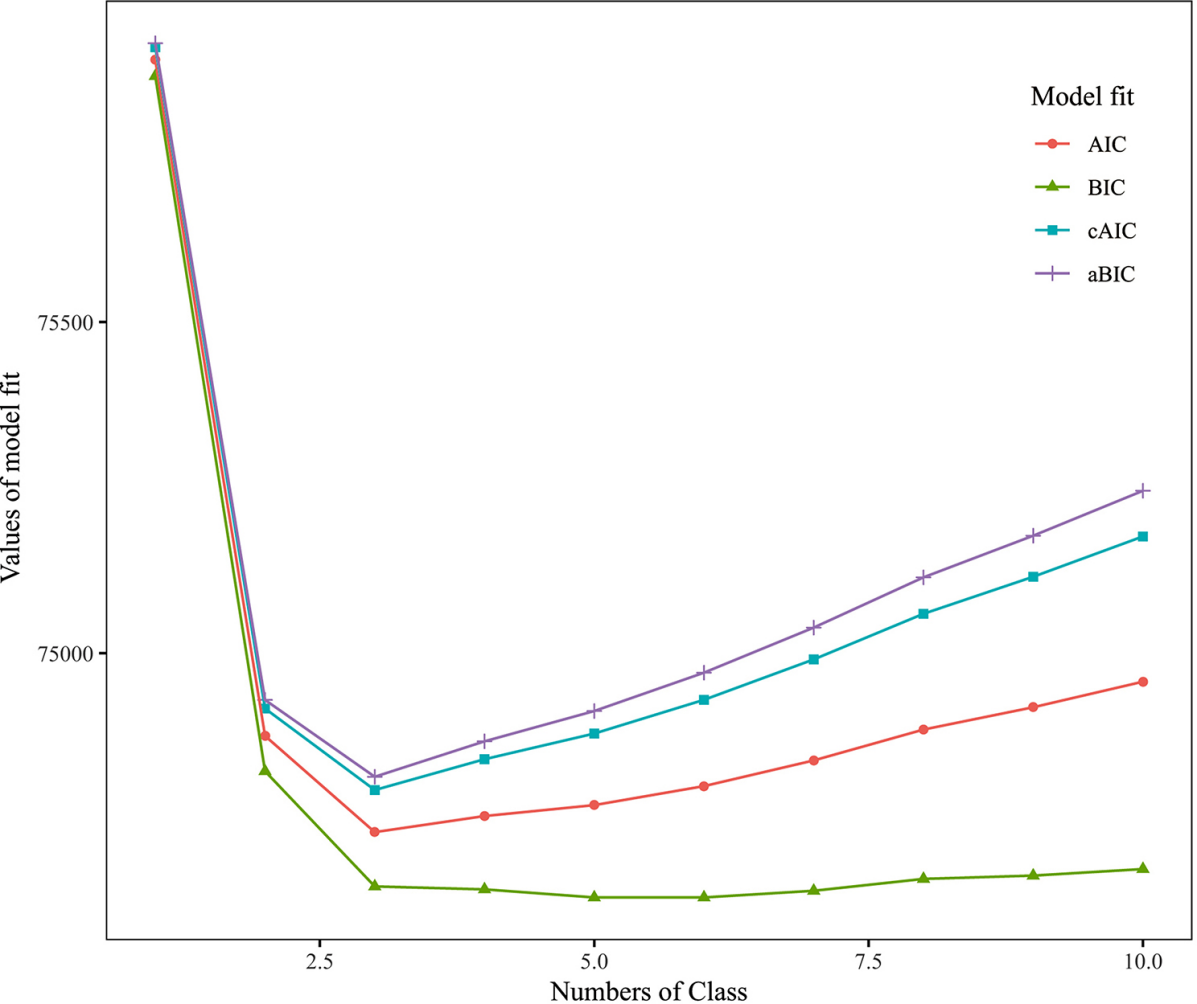
Statistical metrics for model selection

**Fig. 2:**
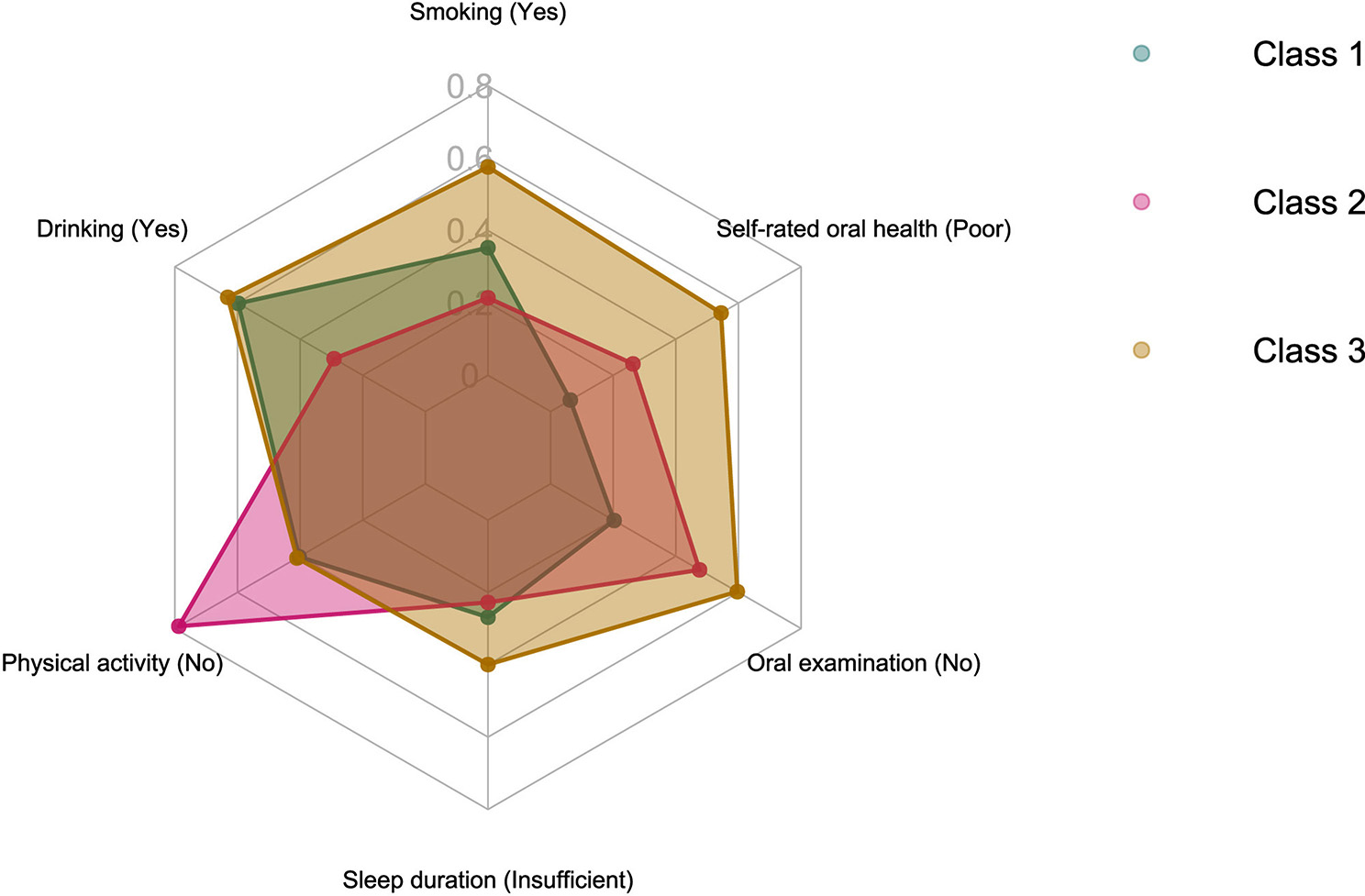
The distribution of latent classes and conditional probability for 6 lifestyles

In the fit results of 2–10 models, all model assessment indicators reached a minimum at 3 classes (Fig. 1). Considering interpretability of the results, 3 classes were selected as the optimal number of latent classes of lifestyles. Latent classes of lifestyles were tagged based on the conditional probability of model fitting (Fig. 2). The ‘Class 1’ exhibited a prevalence of oral examination (75%), favorable self-rated oral health (92%), and engagement in physical activity (60%). The ‘Class 2’ displayed the lowest frequencies of alcohol consumption (64%) and smoking (73%), yet registered the highest incidence of physical inactivity (98%). The ‘Class 3’ demonstrated a propensity for smoking (72%), alcohol consumption (78%), shorter sleep duration (50%), lack of oral examinations (75%), and suboptimal self-rated oral health (68%).

### Characteristics of the latent class of lifestyle

**Fig. 3:**
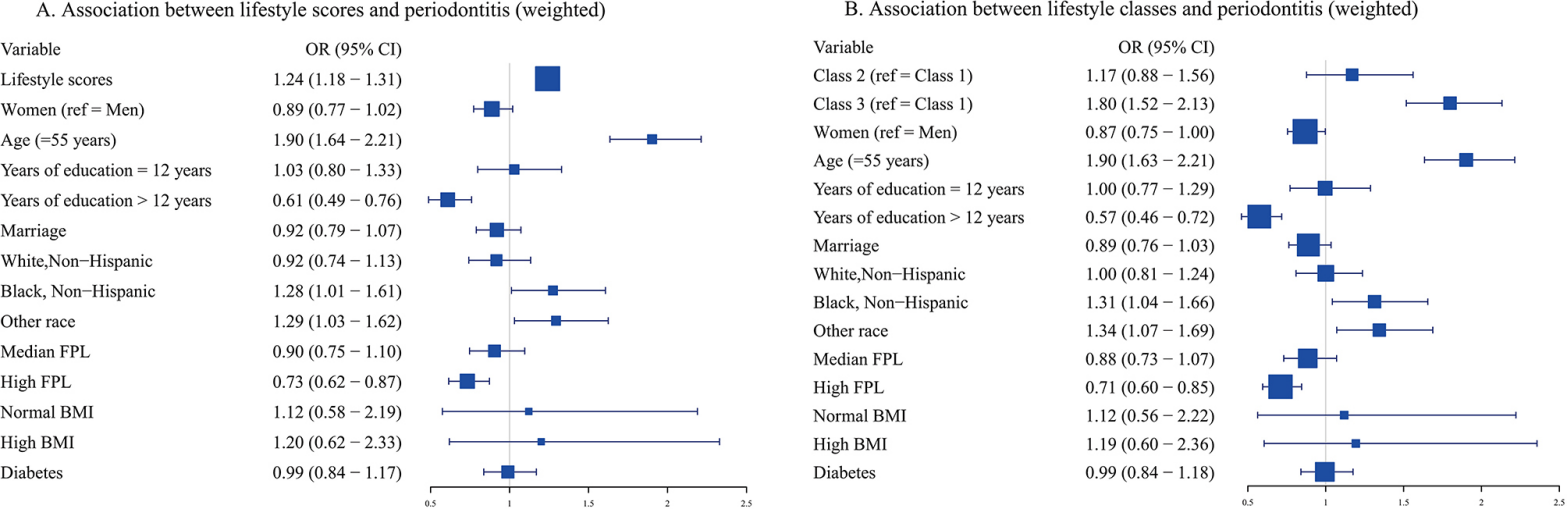
Forest plot for sociodemographic characteristics of latent lifestyle classes based on weighted logistic regression model. (**A**) Sociodemographic characteristics of ‘Class 2’ (Ref = ‘Class 1’). (**B**) Sociodemographic characteristics of ‘Class 3’ (Ref = ‘Class 1’)

Figure 3 lists the sociodemographic details of the several latent classes of lifestyle. Participants in the “Class 2” group were more likely to be older, female, and less educated than those in the “Class 1” group, as well as to have lower levels of poverty. The “Class 3” class had a higher likelihood of being younger, having fewer years of education, being unmarried, and having a lower poverty level.

### Association of lifestyle latent class and scores with periodontitis

**Fig. 4:**
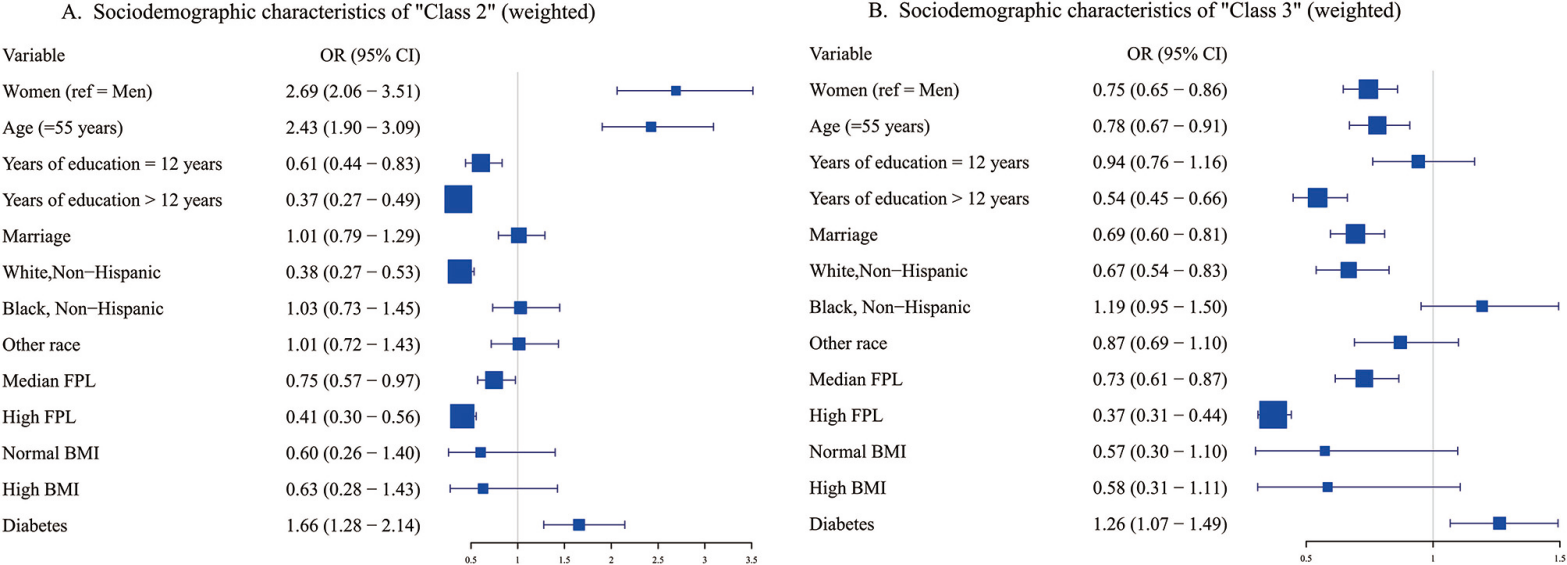
Forest plot for the association between lifestyle and periodontitis based on weighted ordinal logistic regression model. (**A**) The association between lifestyle scores and periodontitis. (**B**) The association between lifestyle classes and periodontitis

Figure 4 illustrates how periodontitis and lifestyle are related. Model 1 assessed the association between periodontitis and poor lifestyle scores. The findings show that participants were more likely to develop periodontitis for each unhealthy lifestyle they increased (OR = 1.24, 95% CI: 1.18–1.31), after controlling for sex, age, marital status, education, race, poverty level, BMI, and diabetes. In model 2, the association between lifestyle latent classes and periodontitis was assessed. The results imply that the risk of periodontitis increased by 80% (95% CI: 1.52–2.13) in the ‘Class 3’ compared to the ‘Class 1’.

## Discussion

In this study, we identified 3 lifestyle latent classes based on 6 lifestyle indicators in NHANES: ‘Class 1’ (52%), ‘Class 2’ (13%), and ‘Class 3’ (35%). By conducting the LCA, participants who maintain lifestyles variously could be distinguished and classified between healthy and poor in aggregation. In addition, current evidence ameliorates the knowledge of association between lifestyle and periodontitis by indicating that aggregated poor lifestyle were strongly associated with the severity of periodontitis. These results may improve the understanding of lifestyle clusters and provide an important reference for the development of interventions for periodontitis.

Our study showed that poor lifestyle clusters were associated with age, years of education, and poverty level. Our findings are consistent with previous studies, and the results suggest that different lifestyle classes may be more associated with social environments that may arouse attitudes and practices toward health behaviors, especially physical activity, etc [27, 28]. For instance, within our study, individuals who were non-smokers and abstained from alcohol consumption while also lacking physical activity were found to be more prevalent in ‘Class 2’. Furthermore, compared to individuals in ‘Class 1’, those in ‘Class 2’ were more likely to be women. Therefore, lifestyle education and intervention programs for periodontitis control need to take these underlying characteristic differences into account.

Although it is clear that people who have more school years are more likely to lead healthier lifestyles, these connections are not always causal. A study in the Philippines with 1064 participants found that health literacy rather than education level encourages a better lifestyle [29]. In addition, a UK study that included 9003 participants showed that health literacy did not increase with years of education after educational reform [30]. Hence, to raise the population’s health literacy and thereby lower the risk of periodontitis, proper intervention programs based on years of education are required when undertaking lifestyle interventions.

Our study also discovered a significant link between unhealthy lifestyle ratings and periodontitis, which is in line with findings from earlier studies [6]. Nevertheless, a lot of useful information was lost in earlier research; our study altered this. The compromise of valid information stems from the amalgamation of distinct unhealthy lifestyles (such as smoking and drinking) into a singular unhealthy count. In other words, participants reporting the same lifestyle as ‘1’ may encompass both smoking and drinking, each potentially eliciting distinct health effects. This blending of diverse health behaviors undermines the discernment of differential benefits across various health lifestyles. In this study, we could clarify that different lifestyle classes were significantly associated with periodontitis.

In our analysis, the “Class 3”, which included several bad lifestyle choices, significantly increased the development of periodontitis. These individuals were more likely to smoke, drink alcohol, have shorter sleep duration, have oral examinations, and report poor self-rated health. Smoking and alcohol consumption are known risk factors. And the association between sleep duration and periodontitis is gradually being explored. Some findings suggest that either too long or too short a sleep duration increases the risk of adverse health outcomes, such as death, cardiovascular disease, diabetes, and obesity [31, 32]. And these adverse health outcomes may further exacerbate the risk of periodontitis [33]. In addition, participants in the ‘Class 3’ rated themselves as having poor oral health but were reluctant to go for oral examinations, suggesting that this class is weak in health awareness and needs more attention. Thus, a combination of lifestyle aggregation and underlying characteristics is needed to develop appropriate public health policies to reduce the risk of periodontitis.

The practice of lifestyle medicine, such as quitting smoking and drinking, is an important therapy method for periodontitis, which dental health explores and reveals. These discoveries have significant consequences for public health. Furthermore, our discoveries can be effectively incorporated into the S3 level guideline for the treatment of periodontitis. By the guideline’s recommendations, oral hygiene practices are applied consistently throughout the phases of periodontal treatment. A favorable lifestyle is particularly crucial during steps 1 (oral hygiene instruction) and 4 (supportive periodontal care) of periodontitis treatment [34, 35]. Our findings facilitate a more precise identification of demographic characteristics within the population, enabling targeted lifestyle promotions to enhance oral hygiene. For instance, placing emphasis on physical activity rather than endorsing smoking and alcohol restrictions for participants among ‘Class 2.’

Despite the good evidence on lifestyle studies for periodontitis interventions, dental practitioners generally lack expertise in lifestyle interventions that have not been well addressed in past dental education [36]. Our findings and the current situation described above suggest the need to prioritize the development of healthy lifestyle programs in the dental office as a ‘first-line treatment’ for periodontitis [37].

This study is one of the first, as far as we are aware, to examine the association between latent lifestyle patterns and periodontitis. However, there are some limitations to this study. First off, since this research was cross-sectional, it’s difficult to determine whether a certain way of living causes periodontitis. Second, we were unable to determine the effect of lifestyle modifications on periodontitis. Because the data on smoking, alcohol intake, and exercise were an evaluation of recent status, it was not possible to determine the relevance of alcohol and smoking cessation on periodontitis. Third, it is crucial to recognize the potential overlap between measures of oral examination and self-assessment of oral health, potentially influencing statistical outcomes. However, these effects are manageable for several reasons. (1) Oral examination and self-rated oral health represent distinct dimensions of measurement; the former is an objective health behavior, and the latter is a subjective health evaluation. (2) LCA can address the impact of overlapping variables resulting from the classification of individuals, provided that local independence is satisfied during these lifestyle measurements. LCA, to a certain extent, assists in mitigating the impact of overlapping variables. Fourth, even if the survey was done face-to-face by trained professionals, the impact of measurement error cannot be entirely disregarded. Furthermore, the use of binary numbers to represent lifestyle might impose considerable restrictions because severe activity and moderate exercise may have similar MET scores but different consequences. In order to categorize lifestyles and determine whether there is a link between them and periodontitis, future studies must use more objective lifestyle metrics.

## Conclusion

In conclusion, we discovered two classes of bad lifestyle patterns—“Class 2” and “Class 3”—that are linked to an increased risk of periodontitis. These associations may offer pertinent cues for devising lifestyle promotion plans and could potentially contribute to formulating etiological hypotheses in accordance with the S3 level guideline for the treatment of periodontitis.

### Electronic supplementary material

Below is the link to the electronic supplementary material.

Supplementary Material 1

## Data Availability

This study used public data at https://www.cdc.gov/nchs/nhanes/index.htm.

## References

[1] Slots J. Periodontitis: facts, fallacies and the future. Periodontol 2000 2017, 75(1):7–23. 10.1111/prd.12221 28758294

[2] Eke PI, Borgnakke WS, Genco RJ (2020). Recent epidemiologic trends in periodontitis in the USA. Periodontol 2000.

[3] Irwandi RA, Chiesa ST, Hajishengallis G, Papayannopoulos V, Deanfield JE, D’Aiuto F (2022). The roles of neutrophils linking Periodontitis and Atherosclerotic Cardiovascular diseases. Front Immunol.

[4] Teles F, Collman RG, Mominkhan D, Wang Y (2022). Viruses, periodontitis, and comorbidities. Periodontol 2000.

[5] Roguljic M, Vuckovic M, Gelemanovic A, Kovacevic K, Oreskovic J, Radic M, Bozic D, Radic J. Risk factors of severe periodontitis in kidney transplant recipients: a case-control study. J Periodontol 2023. 10.1002/JPER.22-0351 36700464

[6] Iwasaki M, Borgnakke WS, Ogawa H, Yamaga T, Sato M, Minagawa K, Ansai T, Yoshihara A, Miyazaki H (2018). Effect of lifestyle on 6-year periodontitis incidence or progression and tooth loss in older adults. J Clin Periodontol.

[7] Bartold PM (2018). Lifestyle and periodontitis: the emergence of personalized periodontics. Periodontol 2000.

[8] Chapple IL, Bouchard P, Cagetti MG, Campus G, Carra MC, Cocco F, Nibali L, Hujoel P, Laine ML, Lingstrom P (2017). Interaction of lifestyle, behaviour or systemic diseases with dental caries and periodontal diseases: consensus report of group 2 of the joint EFP/ORCA workshop on the boundaries between caries and periodontal diseases. J Clin Periodontol.

[9] Loef M, Walach H (2012). The combined effects of healthy lifestyle behaviors on all cause mortality: a systematic review and meta-analysis. Prev Med.

[10] Sinha P, Calfee CS, Delucchi KL (2021). Practitioner’s guide to latent class analysis: methodological considerations and common pitfalls. Crit Care Med.

[11] Ghanbari J, Mohammadpoorasl A, Jahangiry L, Farhangi MA, Amirzadeh J, Ponnet K (2018). Subgroups of lifestyle patterns among hypertension patients: a latent-class analysis. BMC Med Res Methodol.

[12] Nudelman G, Yakubovich S (2022). Patterns of health lifestyle behaviours: findings from a representative sample of Israel. BMC Public Health.

[13] Zhang YB, Chen C, Pan XF, Guo J, Li Y, Franco OH, Liu G, Pan A (2021). Associations of healthy lifestyle and socioeconomic status with mortality and incident cardiovascular disease: two prospective cohort studies. BMJ.

[14] Ahluwalia N, Dwyer J, Terry A, Moshfegh A, Johnson C (2016). Update on NHANES Dietary Data: Focus on Collection, Release, Analytical considerations, and uses to inform Public Policy. Adv Nutr.

[15] Paulose-Ram R, Graber JE, Woodwell D, Ahluwalia N (2021). The National Health and Nutrition Examination Survey (NHANES), 2021–2022: Adapting Data Collection in a COVID-19 environment. Am J Public Health.

[16] Li X, Wang L, Yang L, Liu X, Liu H, Mu Y (2024). The association between plain water intake and periodontitis in the population aged over 45: a cross-sectional study based on NHANES 2009–2014. BMC Oral Health.

[17] Agnew MD, Pettifor H, Wilson C (2023). Lifestyle, an integrative concept: cross-disciplinary insights for low-carbon research. WIRE Energy Environ.

[18] Chu NM, Hong J, Harasemiw O, Chen X, Fowler KJ, Dasgupta I, Bohm C, Segev DL, McAdams-DeMarco MA (2022). Global Renal Exercise N: chronic kidney disease, physical activity and cognitive function in older adults-results from the National Health and Nutrition Examination Survey (2011–2014). Nephrol Dial Transpl.

[19] National Health and Nutrition Examination. Survey 2011–2012 Data Documentation, Codebook, and Frequencies: Physical Acitivty (PAQ_G) [ https://wwwn.cdc.gov/Nchs/Nhanes/2011-2012/PAQ_G.htm].

[20] Eke PI, Dye BA, Wei L, Slade GD, Thornton-Evans GO, Borgnakke WS, Taylor GW, Page RC, Beck JD, Genco RJ (2015). Update on prevalence of periodontitis in adults in the United States: NHANES 2009 to 2012. J Periodontol.

[21] Rutherford ER, Vandelanotte C, Chapman J, To QG (2022). Associations between depression, domain-specific physical activity, and BMI among US adults: NHANES 2011–2014 cross-sectional data. BMC Public Health.

[22] Mainous AG, Tanner RJ, Jo A, Anton SD (2016). Prevalence of prediabetes and abdominal obesity among healthy-weight adults: 18-Year Trend. Ann Fam Med.

[23] Fang Y, Dong Z, Huang T, Wang L, Fan W, Wang B, Yang Q, Xu M, Li D, Fang Y (2023). The role of socioeconomic status and oxidative balance score in erectile dysfunction: a cross-sectional study. Heliyon.

[24] Stanley A, Schuna J, Yang S, Kennedy S, Heo M, Wong M, Shepherd J, Heymsfield SB (2020). Distinct phenotypic characteristics of normal-weight adults at risk of developing cardiovascular and metabolic diseases. Am J Clin Nutr.

[25] Liu C, He L, Li Y, Yang A, Zhang K, Luo B (2023). Diabetes risk among US adults with different socioeconomic status and behavioral lifestyles: evidence from the National Health and Nutrition Examination Survey. Front Public Health.

[26] MacLean E, Dendukuri N (2021). Latent class analysis and the need for Clear Reporting of methods. Clin Infect Dis.

[27] Hernandez EM, Margolis R, Hummer RA (2018). Educational and gender differences in Health Behavior Changes after a Gateway diagnosis. J Aging Health.

[28] Chuang YC, Chuang KY (2008). Gender differences in relationships between social capital and individual smoking and drinking behavior in Taiwan. Soc Sci Med.

[29] Hoffmann R, Lutz SU (2019). The health knowledge mechanism: evidence on the link between education and health lifestyle in the Philippines. Eur J Health Econ.

[30] Johnston DW, Lordan G, Shields MA, Suziedelyte A (2015). Education and health knowledge: evidence from UK compulsory schooling reform. Soc Sci Med.

[31] Shan Z, Ma H, Xie M, Yan P, Guo Y, Bao W, Rong Y, Jackson CL, Hu FB, Liu L (2015). Sleep duration and risk of type 2 diabetes: a meta-analysis of prospective studies. Diabetes Care.

[32] Yin J, Jin X, Shan Z, Li S, Huang H, Li P, Peng X, Peng Z, Yu K, Bao W et al. Relationship of Sleep Duration with all-cause Mortality and Cardiovascular events: a systematic review and dose-response Meta-analysis of prospective cohort studies. J Am Heart Assoc 2017, 6(9). 10.1161/JAHA.117.005947 PMC5634263 28889101

[33] Han DH, Kim MS, Kim S, Yoo JW, Shen JJ (2022). Sleep time and duration are associated with periodontitis in a representative sample of koreans. J Periodontol.

[34] Sanz M, Herrera D, Kebschull M, Chapple I, Jepsen S, Beglundh T, Sculean A, Tonetti MS, Participants EFPW, Methodological C (2020). Treatment of stage I-III periodontitis-the EFP S3 level clinical practice guideline. J Clin Periodontol.

[35] Herrera D, Sanz M, Kebschull M, Jepsen S, Sculean A, Berglundh T, Papapanou PN, Chapple I, Tonetti MS, Participants EFPW (2022). Treatment of stage IV periodontitis: the EFP S3 level clinical practice guideline. J Clin Periodontol.

[36] Albert D, Ward A (2012). Tobacco cessation in the dental office. Dent Clin North Am.

[37] Fiorini T, Musskopf ML, Oppermann RV, Susin C (2014). Is there a positive effect of smoking cessation on periodontal health? A systematic review. J Periodontol.

