# Supplementary material for: Association between modifiable lifestyle pattern and periodontitis: a cross-sectional study based on NHANES
Source: BMC Oral Health. 2024 May 21;24:591. doi: 10.1186/s12903-024-04207-8 (PMC11110925; doi:10.1186/s12903-024-04207-8)
Supplement: Supplementary file 1 — Supplementary Material 1 [file 12903_2024_4207_MOESM1_ESM.doc]

Additional files：STROBE Statement—Checklist of items that should be included in reports of ***cross-sectional studies***

|  | Item No | Recommendation | Page No. Relevant text from manuscrip |  |  |
| --- | --- | --- | --- | --- | --- |
| **Title and abstract** | 1 | (*a*) Indicate the study’s design with a commonly used term in the title or the abstract | Complete |  |  |
|  |  | (*b*) Provide in the abstract an informative and balanced summary of what was done and what was found | Complete |  |  |
| Introduction | | |  |  |  |
| Background/rationale | 2 | Explain the scientific background and rationale for the investigation being reported | Complete-Introduction paragraphs 1-3 |  |  |
| Objectives | 3 | State specific objectives, including any prespecified hypotheses | Complete-Introduction paragraphs 4 |  |  |
| Methods | | |  |  |  |
| Study design | 4 | Present key elements of study design early in the paper | Complete-Methods subheading “Data source” |  |  |
| Setting | 5 | Describe the setting, locations, and relevant dates, including periods of recruitment, exposure, follow-up, and data collection | Complete-Methods subheading “Data source” |  |  |
| Participants | 6 | (*a*) Give the eligibility criteria, and the sources and methods of selection of participants | Complete-Methods subheading “Data source” |  |  |
| Variables | 7 | Clearly define all outcomes, exposures, predictors, potential confounders, and effect modifiers. Give diagnostic criteria, if applicable | Complete-Methods subheading “Variables” |  |  |
| Data sources/ measurement | 8* | For each variable of interest, give sources of data and details of methods of assessment (measurement). Describe comparability of assessment methods if there is more than one group | Complete-Methods subheading “Data source” |  |  |
| Bias | 9 | Describe any efforts to address potential sources of bias | Complete-Methods subheading “Statistical analysis” |  |  |
| Study size | 10 | Explain how the study size was arrived at | Complete-Methods subheading “Data source” |  |  |
| Quantitative variables | 11 | Explain how quantitative variables were handled in the analyses. If applicable, describe which groupings were chosen and why | Complete-Methods subheading “Variables” |  |  |
| Statistical methods | 12 | (*a*) Describe all statistical methods, including those used to control for confounding | Complete-Methods subheading “Statistical analysis” |  |  |
|  |  | (*b*) Describe any methods used to examine subgroups and interactions | Complete-Methods subheading “Statistical analysis” |  |  |
|  |  | (*c*) Explain how missing data were addressed | Complete-Methods subheading “Statistical analysis” |  |  |
|  |  | (*d*) If applicable, describe analytical methods taking account of sampling strategy | Complete-Methods subheading “Data source” |  |  |
|  |  | (*e*) Describe any sensitivity analyses | NA |  |  |
| Results | | |  |  |  |
| Participants | 13* | (a) Report numbers of individuals at each stage of study—eg numbers potentially eligible, examined for eligibility, confirmed eligible, included in the study, completing follow-up, and analysed | Complete-Results subheading “Sample characteristics” |  |  |
|  |  | (b) Give reasons for non-participation at each stage | Not applied |  |  |
|  |  | (c) Consider use of a flow diagram | NA |  |  |
| Descriptive data | 14* | (a) Give characteristics of study participants (eg demographic, clinical, social) and information on exposures and potential confounders | Complete-Results subheading “Sample characteristics” |  |  |
|  |  | (b) Indicate number of participants with missing data for each variable of interest | NA |  |  |
| Outcome data | 15* | Report numbers of outcome events or summary measures | Complete-Results subheading “Sample characteristics” |  |  |
| Main results | 16 | (*a*) Give unadjusted estimates and, if applicable, confounder-adjusted estimates and their precision (eg, 95% confidence interval). Make clear which confounders were adjusted for and why they were included | Complete-Results |  |  |
|  |  | (*b*) Report category boundaries when continuous variables were categorized | Complete-Results |  |  |
|  |  | (*c*) If relevant, consider translating estimates of relative risk into absolute risk for a meaningful time period | NA |  |  |
| Other analyses | 17 | Report other analyses done—eg analyses of subgroups and interactions, and sensitivity analyses | NA |  |  |
| Discussion | | |  |  |  |
| Key results | 18 | Summarise key results with reference to study objectives | Complete- Discussion paragraphs 1 |  |  |
| Limitations | 19 | Discuss limitations of the study, taking into account sources of potential bias or imprecision. Discuss both direction and magnitude of any potential bias | Complete- Discussion paragraphs 7 |  |  |
| Interpretation | 20 | Give a cautious overall interpretation of results considering objectives, limitations, multiplicity of analyses, results from similar studies, and other relevant evidence | Complete- Discussion paragraphs 2-6 |  |  |
| Generalisability | 21 | Discuss the generalisability (external validity) of the study results | Complete- Discussion paragraphs 7 |  |  |
| Other information | | |  |  |  |
| Funding | 22 | Give the source of funding and the role of the funders for the present study and, if applicable, for the original study on which the present article is based | Complete |  |  |

*Give information separately for exposed and unexposed groups.

**Note:** An Explanation and Elaboration article discusses each checklist item and gives methodological background and published examples of transparent reporting. The STROBE checklist is best used in conjunction with this article (freely available on the Web sites of PLoS Medicine at http://www.plosmedicine.org/, Annals of Internal Medicine at http://www.annals.org/, and Epidemiology at http://www.epidem.com/). Information on the STROBE Initiative is available at www.strobe-statement.org.
